# Obesity and Overweight Prevalence among a Mississippi Low-Income Preschool Population: A Five-Year Comparison

**DOI:** 10.5402/2011/270464

**Published:** 2011-09-18

**Authors:** Bonnie L. Harbaugh, Jerome R. Kolbo, Elaine F. Molaison, Geoffrey M. Hudson, Lei Zhang, Danyell Wells

**Affiliations:** ^1^School of Nursing, College of Health, University of Southern Mississippi, 118 College Drive, Box 5095, Hattiesburg, MS 39406-0001, USA; ^2^School of Social Work, College of Health, University of Southern Mississippi, 118 College Drive, Box 5114, Hattiesburg, MS 39406-0001, USA; ^3^Department of Nutrition and Food Systems, College of Health, University of Southern Mississippi, 118 College Drive, Box 5172, Hattiesburg, MS 39406-0001, USA; ^4^School of Human Performance and Recreation, College of Health, University of Southern Mississippi, 118 College Drive, Box 5142, Hattiesburg, MS 39406-0001, USA; ^5^Office of Health Data and Research, Mississippi State Department of Health, 570 E. Woodrow Wilson, Jackson, MS 39215-1700, USA

## Abstract

*Purpose*. This study determined 2010 rates of overweight/obesity in a representative sample of low-income preschoolers in Mississippi, USA and compared rates between 2005 (*N* = 1250) and 2010 (*N* = 1765). *Significance*. Obesity is a significant global health issue because of its well-established negative health consequences. Child obesity is a concern due to risk of early-onset obesity-related illnesses and the longevity of lifetime exposure to those illnesses. *Methods*. Identical measures were used in 2005 and 2010 with complex-stratified sampling designs. *Results*. Chi-square tests revealed that overall obesity/overweight rates between 2005 (20.6%/17.9%) and 2010 (20.8%/17.0%) had not changed significantly for the samples as a whole, nor by gender or race. Age group comparisons indicated a significant decline in obesity rates of 3 year olds (20.3% in 2005, reduced to 13.1% in 2010, *P* = 0.035). These findings mimic the trend toward stabilization of obesity rates noted in national low-income preschool populations.

## 1. Introduction

Child obesity is a significant global health issue of concern to nurses because of its well-established negative health consequences [1–10]. Obesity in preschoolers (ages 2–5 years) is a serious health concern due to the increased risk for early onset of obesity-related illnesses and the increased longevity of lifetime exposure to those illnesses [11]. Epidemiological evidence of the prevalence and trends of childhood obesity provides essential baseline data for nurses involved in teaching, planning, and intervening against this important health risk. 

While worldwide estimates indicate that preschool obesity is on the rise [12], recent USA studies indicate that national rates of preschool obesity have stabilized, halting the alarming rises experienced in the previous 10 years. For instance, in a national USA multistage probability sampling of preschoolers, obesity rates dropped from 13.9% in 2003-2004 to 11% in 2005-2006 and to 10.4% in 2007-2008, a level last seen in 1999-2000 [13]. This stabilization trend also holds true for national USA samples of low-income preschoolers from federally funded health programs, including Head Start, whose obesity rates remained stable from 2003 (14.5%) to 2008 (14.6%) [14, 15]. Obesity rates for subgroups of low-income preschoolers had stabilized for non-Hispanic White (12.6%), non-Hispanic Black (11.8%), and Hispanic (18.5%) preschoolers, while rates continued to rise slightly for Native American or Alaska native (21.2%) preschoolers [14]. 

Geographic distribution of obesity in adults and children remains highest in the South US, and in high-poverty states [16]. Mississippi, a southern state with the highest level of poverty [17], had the highest adult obesity rates in the USA at 33.8% [16, 18]. In addition, 44.4% of Mississippi children aged 10–17 years of age self-reported as overweight or obese in the 2007 National Survey of Children's Health [19] versus the national rate of 31.6%. More recent measured estimates of Mississippi Kindergarten through 12th grade (K-12) obesity and overweight rates in 2009 indicated a combined rate of 23.9%, not significantly different from the 2007 combined rate of 23.5% [20].

 In 2005, approximately 39% of Mississippi Head Start (federally funded low-income school-readiness program) preschoolers were obese or overweight [21], compared to a combined rate of 26% for low-income preschoolers in a USA national sample in a similar time period [22]. In 2009, the pediatric nutrition surveillance system reported 13.9% of low-income preschoolers in Mississippi aged 2–5 years were obese [15]. 

Though national preschool weight trends appear to be stabilizing, obesity levels still remain high in racial subgroups and in low-income preschoolers when compared to national rates of all USA preschoolers and to rates of non-Hispanic White preschoolers [13]. Some subgroups of preschoolers remain at high risk for obesity-related illnesses and are in special need of weight trend monitoring as well as nursing interventions to reduce overweight and obesity. For example, national obesity rates were higher among Latinos (14.2%) and non-Hispanic Blacks (11.4%) than non-Hispanic White preschoolers (9.1%) [13]. The trend for obesity in low-income preschoolers also showed Hispanics had higher rates with 17.9%, followed by non-Hispanic White (12.3%) and non-Hispanic Blacks (11.9%) [15]. Native American and Alaskan native children remain the group with the highest rates of obesity [15].

Regularly sequenced weight trend analyses are necessary for nurses to track disease surveillance and for health policy planning, intervention, and evaluation. This study assessed the heights and weights of a representative sample of Mississippi Head Start preschoolers in 2010 and compared obesity and overweight rates with those of an identical assessment in 2005 [21]. 

## 2. Materials and Methods

Identical standardized measurement procedures were used in both the 2010 and the 2005 [21] cross-sectional studies. The only exception was in the timing of data collection. The 2010 data were collected in March/May of 2010, while the 2005 data were collected in October/November of 2005. These studies used a two-stage stratified randomized probability sample, designed to yield self-weighting, which gave every eligible child an equal chance of being selected. This strategy improved the precision of the estimates, and the process produced a sample estimated to accurately represent the entire Head Start population in Mississippi. Each child was assigned a base weight equal to the inverse of the probability of selection for that child. Adjustments were made to initial weights to remove bias from the estimates and reduce the variability, including adjusting for students who were absent on the day of the data collection, students who did not receive parental permission, or students who refused to participate. A second adjustment made was poststratification, where the weighted sample distribution was aligned with population totals by gender. 

In 2010, classrooms of preschoolers were sampled randomly from a frame of 23,858 students in 219 Head Start Centers (all Head Start Centers and students in Mississippi). In the first stage, 36 centers were systematically drawn with probability proportional to the enrollment in the center. In the second stage, 2–5 classes at each center were selected using equal probability systemic sampling, using randomly generated numbers based on the maximum numbers of classrooms at each center. All preschoolers in each of the selected classrooms were asked to participate in the study. In 2010, a total of 1,765 preschoolers from the 2,167 sampled students (81%) participated. The center response rate was 100% (36 participating centers/36 sampled centers). Thus, the overall response rate for 2010 was 81% (product of center response rate and student response rate), which exceeded 60%, the hurdle rate set by the Centers for Disease Control and Prevention in order for weighted data to be used in analysis [23]. Similarly, in 2005, a total of 1,250 preschoolers from the 2,009 eligible sampled students (60.3%) participated [21], which also met the hurdle rate. By using weighted percentage data in analyses, results from the 2005 and 2010 surveys can be generalized to all Mississippi Head Start preschoolers.

Both the 2010 and 2005 studies were approved by the institutional review board of The University of Southern Mississippi. Once the centers agreed to participate, and classes were selected, measuring equipment (UC 321 Pro Fit Precision Digital Scales (AD Engineering, San Jose, Calif) and Seca 214 Road Rod Portable Stadiometers (Seca Corp, Hanover, Md)), Optiscan forms (Optiscan, Inc., Phoeniz, Ariz) for gathering data, and consent forms were supplied to the centers by the researchers. Each center designated a health data collector (some were center health coordinators, some were registered nurses) who attended a half-day training session, which provided information on the project, the protocol, and the use of the measuring equipment to ensure consistency in measurement. Reliability checks were performed with the trainer during the training session. 

Two days before beginning data collection, the health data collector read a prepared paragraph containing information about the study to the children in the selected classes. Each child was given a consent form to take home to guardians or parents. The consent form described the purpose of the study, risks and benefits, and the procedures the health data collector would follow to obtain heights and weights. There were no consequences for nonparticipation, nor were there rewards for participation.

Children were measured individually and the time of day children were measured remained flexible for each center to accommodate teacher and health data collector schedules. Children were briefly excused to go to the health/nurse's office or a selected room for measurement. No other students were present during the measurement; only the health data collector and the student were present during measurement. The measurement protocol required that the stadiometers and weight scales be placed on a hard smooth surface in a private area. The scales were tared to zero before use and after every student. Children were asked to remove hats, belts, heavy jewelry, jackets, and shoes. Height was measured in inches; weight in pounds. Height and weight, rounded up or down to the nearest whole inch or pound, were recorded on an Optiscan form, along with age, gender, date of birth, racial or ethnic background, and the preschool code number. Data forms were mailed back to the researchers.

Completed records (2010 *N* = 1,765; 2005 *N* = 1,250) of measured height and weight as well as gender and age were used to calculate body mass index (BMI) for each child. BMI was calculated for each preschooler based on the height (in meters) and weight (in kilograms). The height in feet/inches and weight in pounds were first converted to meters and kilograms, and then the BMI was calculated using the SAS program (SAS Institute, Cary, NC) g-c-calculate-BIV.sas as follows: BMI = weight (in kilograms)/((height in meters))^2^ [24]. BMI values were screened to ensure that the results were biologically plausible, using the limits established by the Division of Nutrition and Physical Activity, CDC [24, 25]. BMI percentiles using values from the 2000 gender-specific BMI-for-age growth charts [25] were then computed using the SAS program gc-calculate-BIV.sas [24]. Children were classified as underweight (BMI is less than or equal to the 5th percentile), healthy weight (BMI is greater than the 5th but less than the 84th percentile), overweight (BMI is equal to 85th but less than the 95th percentile), or obese (BMI is equal to or greater than the 95th percentile). SUDANN 10.0 (RTI, Research Triangle Park, NC, 2010) was used to calculate summary statistics and to adjust these estimates to account for differences in the complex sampling structure of the survey. Summary statistics included subgroup-specific prevalence estimates and 95% confidence intervals (CIs). Test of significant differences in prevalence estimates within 2010 and between 2010 and 2005 was performed using chi-square tests. 

## 3. Results

For 2010, the sample (*N* = 1765) subsets consisted of 280 (16%) 3 year olds, 868 (49%) 4 year olds, and 612 (35%) 5 year olds; 1,497 (86%) black, 162 (9%) white, 87 Hispanic (4%), and 19 (1%) other races; 942 boys (53%) and 823 girls (47%) (Table 1). Preschoolers were classified by weight status: 4% were underweight; 58% were healthy weight; 17% were overweight; 21% were obese. The 2005 sample was within 2–4% of values for the 2010 sample, with the exception of age. The 2005 sample was younger than the 2010 sample. This was due in part to differences in timing of data collection, and random sampling may have also contributed. Weight status classifications for 2005 were as follows: 3% were underweight; 58% were healthy weight; 18% were overweight; 21% were obese [21]. 

### 3.1. 2010 Prevalence of Obesity and Overweight

**Obesity**

In 2010, the overall obesity rate was 20.8% (Table 2). The obesity prevalence for boys was significantly higher at 22.7% compared to 18.6% for girls (*P* = 0.036) (Table 3). The obesity prevalence for whites (19.0%) was not significantly different from blacks (20.6%) (*P* = 0.486). The obesity prevalence for children 3, 4, and 5 years of age was 13.1%, 22.7%, and 21.6%, respectively, and the differences among the three age groups were significant (*P* = 0.043).

**Overweight**

In the 2010 study, the overall rate of overweight was 17.0% (Table 2). The overweight prevalence for boys (16.0%) and girls (18.2%) was not significantly different (*P* = 0.118) (Table 3). The overweight prevalence for whites (17.8%) was not significantly higher than blacks (16.9%) (*P* = 0.961). The overweight prevalence for children 3, 4, and 5 years of age was 14.0%, 17.0%, and 18.4%, respectively, and differences among the three age groups were not significant (*P* = 0.293).

### 3.2. Comparisons of 2005 and 2010 Findings

**Obesity**

**Figure 1:**
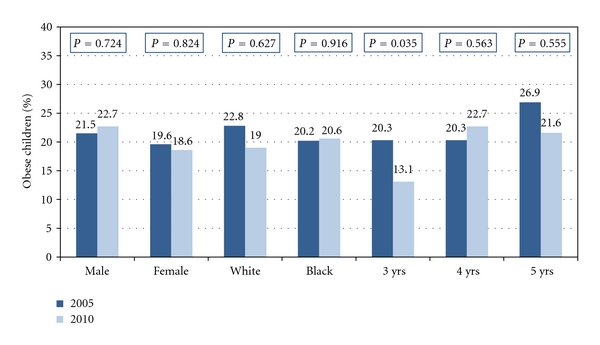
Differences in obesity by gender, race, and age—2005 versus 2010.

The differences in overall obesity prevalence and prevalence by gender, race, gender and race, and age between 2005 and 2010 are presented in Table 2 and in Figure 1. The overall obesity prevalence between 2005 (20.6%) and 2010 (20.8%) was virtually unchanged. The prevalence for males increased from 21.5% in 2005 to 22.7% in 2010 and females decreased from 19.6% to 18.6% during the same period. However, none of the changes were statistically significant. In terms of race, no significant differences were observed among white and black preschoolers between 2005 and 2010. However, there was a significant decrease in obesity prevalence for 3-year old children (*P* = 0.035). The rates changed from 20.3% in 2005 to 13.1% in 2010 for this age group.

**Overweight**

**Figure 2:**
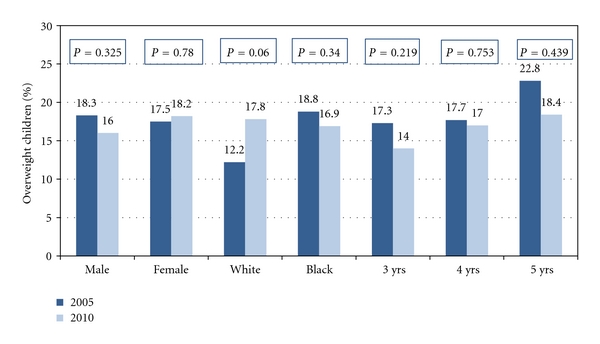
Differences in overweight by gender, race, and age—2005 versus 2010.

The overall overweight prevalence and prevalence by gender, race, gender and race, and age for 2005 and 2010 are presented in Table 2 and in Figure 2. Analyses for overall overweight rates indicated insignificant changes between 2005 (17.9%) and 2010 (17.0%). Further, there were no significant changes between boys (2005: 18.3%; 2010: 16%), girls (2005: 17.5%; 2010: 18.2%), whites (2005: 12.2 %; 2010: 17.9 %), blacks (2005: 18.8%; 2010: 16.9 %), 3 year olds (2005: 17.3%; 2010: 14.0%), 4 year olds (2005: 17.7%; 2010: 17.0 %), or 5 year olds (2005: 22.8%; 2010: 18.4%).

## 4. Discussion

The findings indicate that higher than national obesity and overweight rates for Mississippi low-income preschoolers persist in 2010, yet comparisons between 2005 and 2010 data mimic the trend toward stabilization of overall obesity/overweight rates noted in other US preschool populations [13, 15]. The 2010 results show that males in this study had significantly higher rates of obesity than females. The 2010 obesity rates by race and gender indicated the lowest rates were in white female preschoolers, which makes it likely that low white female rates accounted for most of the differences between males and females. These findings are consistent with those of the 2009 Child and Youth Prevalence of Obesity Study of Mississippi public school students [20], where the lowest rate of obesity was found among the youngest (kindergarten through second grade) white females. In the 2005 study, no significant gender differences in obesity or overweight were found [21]. Likewise, no differences in overweight or obesity were found between black and white preschoolers in 2005, which match findings in the 2010 study. Significant age differences were not found in 2005, but were highest in the 5 year olds, and were about the same in 3 and 4 year olds [21]. In 2010, the highest rates were in the 4 and 5 year olds, with significantly lower rates found in the 3 year olds. Thus, while overall trends are somewhat stable, some of the most positive changes are occurring within gender and race and also within age groups. Given the complexity of the genetic, biological, behavioral, and social contributors to child obesity, particularly in regards to gender [26], further study is needed to examine the differences in between genders and race in terms of exposure and vulnerability to therapeutic and obesogenic environments both at preschool and in the home. A secondary data analysis using regression statistics may provide more insight into interrelationships between obesity/overweight, gender, age, and race. 

Several limitations of this study deal with timing and history. First, Hurricane Katrina occurred about 2 months prior to 2005 data collection, affecting the bottom two-thirds of the state, and it is unknown how much disruption it may have caused in food and eating habits of that cohort of low-income preschoolers. Second, data were collected earlier in the year 2005 (October/November) than in 2010 (March/April) due to timing of funding and the need for 2010 data by policy and law makers. Thus, there are older children and children who had been in Head Start Programs a few months longer represented in the 2010 study. This situation may account for age differences in BMIs. Further research is needed to study seasonal and longitudinal BMI percentile changes in subgroups of Head Start preschoolers in Mississippi that may result from therapeutic teaching, dietary and physical activity interventions experienced within Head Start preschool environments and perhaps transferred to the home environment. 

Another finding of interest among the Mississippi Head Start preschool population was that the prevalence of children from other racial/ethnic backgrounds (mainly Hispanic and Latino) has increased from 2.7% in 2005 [21] to 5.7% in 2010. The increase in Latino and Hispanic children in Mississippi is the result of workers who immigrated into the area to help after Hurricane Katrina, stayed, and began families. In 2010, the obesity prevalence for Head Start preschoolers with races other than white or black was also the highest, at 26.6%. This is an important finding as the Hispanic and Latino populations are likely to continue to increase in Mississippi, which may influence future preschooler weight trends in this state. This increase in Hispanic children in low-income programs is consistent with national findings in the PedNSS 2009 report [15].

Because of the small sample sizes for white children, it should be noted that subgroup analyses by race and gender may not be reliable. Nevertheless, these findings suggest additional research is needed with more heterogeneous preschool populations that include larger samples of different subgroups (white and Latino/Hispanic children) in various settings (e.g., private preschool centers, day care). By doing so, additional insight can be determined as to the roles of gender, race, and income status, as well as the possible impact of different programs and interventions. The additional information can be useful for informing future obesity prevention and weight-normalizing interventions.

Across the State of Mississippi, a range of obesity reduction interventions are being introduced and implemented at the state and school levels [16], which may influence the families of preschoolers, as well as the Head Start teachers and curricula. There was a five-year gap between these two prevalence studies and, as such, detailed environmental and historical changes on prevalence rates could not be observed. Given the high prevalence rates among this low-income population, continued surveillance and intervention are warranted. Future data collection should occur at closer intervals, possibly every other year.

## 5. Conclusions

While Mississippians are more aware of the seriousness of the obesity problem [27] and have made great strides in statewide policies to address obesity in school-aged children, there has not been the same amount of progress for preschool children. Some Mississippi Head Start Centers have begun trying a variety of interventions since 2005, including serving more vegetables and low-fat foods, nutritional consults with overweight and obese children and their families, portion control, fewer movies and sedentary activities, and more supervised physical activity (personal communications, Dr. Peggy Answorth, President, and Nita Norphlet-Thompson, Executive Director of Mississippi Head Start Association, November 30, 2010). However, implementation of these interventions is not yet comprehensive or well standardized. Until systematic interventions and evaluations occur, it is unlikely that Mississippi will experience a significant drop in preschool obesity rates. 

Nurses, with their health promotion focus, leadership skills, and widespread access to children, are ideal interventionists to address child obesity [28] in Mississippi and the nation. The National Association of Pediatric Nurse Practitioners [29], The American Medical Association [30], and the Institute of Medicine [31] have published position statements and clinical guidelines to address obesity in preschoolers that can be utilized by nurses. However, evidence suggests that treating a child after he or she is already overweight or obese is difficult, therefore nurses are urged to participate in early identification and anticipatory guidance to systematically reduce the prevalence of childhood obesity and its devastating comorbidities at the family, community, state, and national levels [28, 29, 32]. This study provides benchmark prevalence data which clearly indicates that nurses in Mississippi need to identify children at risk and to employ evidence-based interventions directed at preventing obesity in the Head Start preschool population. 

## Figures and Tables

**Table 1:** Characteristics of participants.

	2005^a^		2010	
Characteristic	Unweighted count^b^	Weighted percent^c^	Unweighted count^b^	Weighted percent^c^
Gender^d^				
Male	645	51.3	942	53.4
Female	605	48.7	823	46.6
Race/Ethnicity				
White	127	8.9	162	8.6
Black	1,090	88.4	1,497	85.7
Other	33	2.7	106	5.7
Age (years)^e^				
3	432	35.2	280	16.3
4	757	60.2	868	49.1
5	60	5.5	612	34.3

Total	1,250	100	1,765	100

^a^2005 data from Harbaugh et al. [21].

^b^Preweighted frequencies.

^c^Percentages calculated from weighted frequencies.

^d^2005—Data on gender and age were missing for one child.

^e^2010—Five of the sampled students were 6-years-old.

**Table 2:** Percentage of overweight and obesity by characteristics 2005^a^ and 2010.

	Overweight^b^ (%, 95% CI^d^)		Obese^c^ (%, 95% CI^d^)	
Characteristic	2005	2010	2005	2010
Gender				
Male	18.3 (15.3–21.7)	16.0 (13.0–19.5)	21.5 (19.2–24.1)	22.7 (16.1–31.0)
Female	17.5 (14.7–20.8)	18.2 (15.8–20.8)	19.6 (16.4–23.1)	18.6 (12.1–27.5)
Race				
White	12.2 (9.1–16.3)	17.8 (11.8–25.9)	22.8 (17.1–29.8)	19.0 (8.5–37.2)
Black	18.8 (16.4–21.3)	16.9 (14.6–19.5)	20.2 (18.0–22.6)	20.6 (14.7–28.1)
Race/Gender				
White male	8.5 (4.4–15.8)^e^	17.1 (10.0–27.8)^e^	22.2 (12.0–37.5)^e^	22.9 (8.5–48.7)^e^
Black male	19.6 (16.4–23.2)	15.9 (13.0–19.3)	21.3 (18.1–25.0)	22.1 (16.4–29.1)
White female	16.9 (8.4–31.1)^e^	18.5 (10.4–30.8)^e^	23.5 (15.4–34.3)^e^	14.8 (6.2–31.4)^e^
Black female	18.0 (15.2–21.1)	18.1 (15.9–20.5)	19.0 (15.6–22.9)	18.9 (12.2–27.9)
Age (years)				
3	17.3 (14.2–20.9)	14.0 (10.2–18.9)	20.3 (17.1–23.9)	13.1 (8.9–18.9)
4	17.7 (15.0–20.8)	17.0 (14.2–20.3)	20.3 (17.2–23.8)	22.7 (16.0–31.2)
5	22.8 (13.9–35.1)	18.4 (14.3–23.3)	26.9 (16.9–40.0)	21.6 (13.2–33.3)

Total	**17.9** (15.8–20.1)	**17.0** (14.5–19.8)	**20.6** (18.6–22.8)	**20.8** (14.4–29.1)

^a^2005 data from Harbaugh et al. [21].

^b^Body mass index (BMI) ≥85th percentile and <95th percentile for age and gender.

^c^Body mass index (BMI) ≥95th percentile for age and gender.

^d^95% confidence interval.

^e^Sample size is less than 50. The results may not be reliable.

**Table 3:** Differences in overweight and obesity 2010.

	Overweight*	*P*-value	Obesity^†^	*P*-value
Characteristic	% (95% CI)		% (95% CI)	
Gender				
Males	16.0 (13.0–19.5)	0.118	22.7 (16.1–31.0)	0.036
Females	18.2 (15.8–20.8)		18.6 (12.1–27.5)	
Race				
White	17.8 (11.8–25.9)	0.961	19.0 (8.5–37.2)	0.486
Black	16.9 (14.6–19.5)		20.6 (14.7–28.1)	
Age (yrs)				
3	14.0 (10.2–18.9)	0.293	13.1 (8.9–18.9)	0.043
4	17.0 (14.2–20.3)		22.7 (16.0–31.2)	
5	18.4 (14.3–23.3)		21.6 (13.2–33.3)	
Total	17.0 (14.5–19.8)	NA	20.8 (14.4–29.1)	NA

*BMI-for-age ≥85th percentile, <95th percentile for gender.

^†^BMI-for-age ≥95th percentile for gender.
